# Determinants of Successful Human Immunodeficiency Virus Treatment Outcomes: A Linkage of National Data Sources in Malaysia

**DOI:** 10.21315/mjms2023.30.1.15

**Published:** 2023-02-28

**Authors:** Wan Nur Syamimi Wan Mohamad Darani, Xin Wee Chen, Ely Zarina Samsudin, Fadzilah Mohd Nor, Ismawati Ismail

**Affiliations:** 1 Department of Public Health Medicine, Faculty of Medicine, Universiti Teknologi MARA, Selangor, Malaysia; 2 HIV Unit, Kuala Lumpur and Putrajaya Federal Territories Health Department (JKWPKLP), Ministry of Health, Kuala Lumpur, Malaysia; 3 Institute of Medical Molecular Biotechnology (IMMB), Faculty of Medicine, Universiti Teknologi MARA, Selangor, Malaysia; 4 Department of Medical Microbiology and Parasitology, Faculty of Medicine, Universiti Teknologi MARA, Selangor, Malaysia; 5 Integrative Pharmacogenomics Institute (iPROMISE), Universiti Teknologi MARA, Puncak Alam Campus, Selangor, Malaysia

**Keywords:** human immunodeficiency virus (HIV), viral load, antiretroviral therapy

## Abstract

**Background:**

Concerted efforts have been undertaken to reduce the human immunodeficiency virus (HIV) infection by the year 2030 in Malaysia. A situational analysis of the performance of successful HIV treatment and its determinants is vital; however, this information remains scarce. This study aimed to identify the determinants of undetectable viral load among people living with HIV (PLHIV).

**Methods:**

Newly diagnosed HIV cases (*n* = 493) registered under the Malaysia HIV/AIDS-related national databases from June 2018 to December 2019 were studied. The deterministic matching method was applied to link the records in two national databases (at Kuala Lumpur and Putrajaya Federal Territories Health Department, JKWPKLP HIV line-listing database and National AIDS Registry). Successful HIV treatment, an outcome variable, was measured by the undetectable viral load < 200 copies/mL after 1 year of antiretroviral therapy initiation. Logistic regression analysis was applied in the current study.

**Results:**

Results showed that 454/493 (92.2%; 95% confidence interval [CI]: 89.8%, 94.6%) PLHIV had successful HIV treatment. Study participants had a mean (SD) age of 30 (8.10) years old, predominantly male (96.1%) and sexually transmission (99.9%). The multiple logistic regression analysis revealed two significant determinants including the timing of ART initiation (AOR = 3.94; 95% CI: 1.32, 11.70; *P* = 0.014) and establishment of Sexually Transmitted Infection Friendly Clinic (STIFC) (AOR = 3.40; 95% CI: 1.47, 7.85; *P* = 0.004). Non-significant variables included gender, education level, HIV risk exposure, and co-infections of tuberculosis and Hepatitis C.

**Conclusion:**

JKWPKLP is on the right track to achieving universal treatment as a prevention strategy. Reinforcement of early ART initiation and establishment of STIFC are recommended.

## Introduction

Human immunodeficiency virus (HIV) is a widely prevalent and ongoing public health issue globally as well as in Malaysia. At the end of 2020, an estimated 0.7% (0.6%–0.9%) of adults aged 15 years old–49 years old worldwide were living with HIV and HIV prevalence in Malaysia was 0.4% (0.3%–0.4%) (1, 2). The joint United Nations Programme on HIV and AIDS (UNAIDS) and the World Health Organization (WHO) have set new targets to reinvigorate efforts to eliminate AIDS as a public health issue (3). By 2030, the aim is to achieve the ‘95–95–95’ target (also known as the ‘treatment cascade’ of HIV care), with specific goals of having 95% of all HIV-positive people knowing their status, 95% of all HIV-infected people knowing their status on antiretroviral therapy (ART) and 95% of those on ART being virally suppressed (4). Studies revealed that early HIV treatment could improve clinical outcomes, so the use of HIV treatment as prevention (TasP) was recommended as a realistic preventive and control strategy to prevent HIV transmission (4–6). Combination ART is preferred and currently practiced as first-line ART in Malaysia (7).

Two large-scale trials that support TasP (8, 9) in the PLHIV community have shown that maintaining an undetectable HIV viral load of less than 200 copies/mL can successfully prevent HIV transmission. The trials reported that undetectable viral load equals untransmittable HIV infection, also called undetectable equals untransmittable (U = U) concept. By complying with treatment, PLHIV could become virally suppressed and maintain an undetectable HIV viral load with minimal chance of transmitting HIV to others as well as preventing AIDS-defining illness.

Accordingly, numerous studies were conducted on PLHIV to investigate the factors (e.g. age, gender, income level, marital status, HIV mode of transmission/risk exposure, baseline CD4 count and presence of co-infections) associated with viral load suppression. However, the majority of them defined viral load suppression using a broader range of HIV viral load plasma levels, i.e. HIV viral load of < 1,000 copies/mL (10–16). Studies conducted in developed countries (the United States of America and the United Kingdom) with different health care systems, implementation of policy/guidelines and treatment regimens (17, 18) showed evidence of the determinants (e.g. age, race, employment and education status) for successful HIV treatment (undetectable HIV viral load, viral load < 200 copies/mL). In developing countries such as Malaysia, such evidence describing determinants of undetectable HIV viral load is scarce, thus posing a challenge in preventing new HIV infections and ending AIDS.

**Figure 1:**
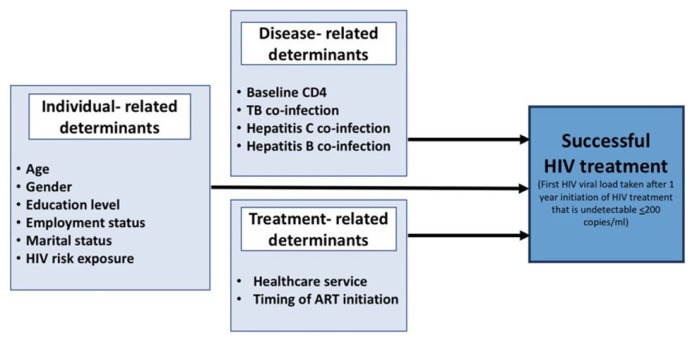
Conceptual framework highlighting individual-, disease- and treatment-related determinants associated with successful HIV treatment in the present study

Besides the individual- and disease-related factors, the treatment-related determinants (Figure 1) associated with successful HIV treatment are equally essential for their modifiable nature. To date, Malaysia lacks evidence-based information with regard to the current healthcare service offered to PLHIV such as the establishment of the Sexually Transmitted Infection Friendly Clinic (STIFC) introduced by the Malaysia Ministry of Health (MOH) in 2015 (19). STIFC was initiated at select government health clinics, where most of them were under the care of the Kuala Lumpur and Putrajaya Federal Territories Health Department (JKWPKLP), around 5 out of 15 health clinics involved with HIV management in JKWPKLP have established STIFC. This STIFC aims to ensure better treatment for PLHIV and provide comprehensive multi-disciplinary management. Nonetheless, recent data associating the STIFC, and successful HIV treatment are limited. Besides, obtaining the information on the timing of ART initiation (17, 18) and Hepatitis B and C co-infection (15) among PLHIV in Malaysia is a key parameter to improve the outcome of PLHIV and subsequent successful treatment.

In order to achieve the national target to reduce HIV infection by 2030, a better understanding of our performance is desirable in attaining successful HIV treatment (undetectable HIV viral load) and its determinants. Besides, the modifiable determinants can be evaluated and compared with the other existing local studies as well as the international studies. These data are crucial to reviewing the status of PLHIV management in the local setting. They can aid in the improvement of our health care system by allowing: i) the public health sector to revise the HIV prevention strategy; ii) clinicians to improve the clinical practice guideline and iii) policymakers to recuperate the health care system, thereby helping achieve the national target to end AIDS by 2030 (20).

This study aimed to determine the successful HIV treatment rate (undetectable viral load < 200 copies/mL) and model its determinants among PLHIV, through establishing linkage across national databases.

## Methods

The current study is etiologic research with a retrospective cohort study design. It analysed the national data sources collected from 15 health clinics managing PLHIV under the catchment of JKWPKLP.

### Study Population and Sampling

The target population in this study was PLHIV living in JKWPKLP. Newly diagnosed HIV cases registered under the Malaysia HIV/AIDS-related national databases from June 2018 to December 2019 were studied. PLHIV who fulfilled the inclusion criteria of the study were recruited and those not meeting the same were excluded. The inclusion criteria were: i) newly diagnosed HIV cases from 1st June 2018 until 31st December 2019; ii) HIV cases notified through Malaysia National AIDS Registry (NAR) from 1st June 2018 until 31st December 2019 and iii) PLHIV aged > 18 years old. Exclusion criteria included: i) PLHIV receiving a monotherapy regime for ART; ii) PLHIV lost to follow-up, deceased, or who did not turn up for viral load testing after one year of ART initiation and iii) non-Malaysian PLHIV.

The sample size was calculated using the OpenEpi Software (21) based on the independent variables listed in the conceptual framework (Figure 1) using the two-proportion formula. Based on a power of 80% (*β* = 0.02) and alpha of 0.05, the required sample size ranged from 187 to 482. The biggest sample size had 482 subjects (with the adjustment of 10% missing responses), by taking the expected detectable difference (odds ratio) of 2.0 and 74% of PLHIV with baseline CD4 < 350 cells/mm^3^ having viral load suppression (12). The observations and informal report by JKWPKLP showed the number of new HIV cases recorded in JKWPKLP each year was around 500 cases; hence, a universal sampling method was applied to select cases fulfilling the inclusion and exclusion criteria.

### Data Source

A data linkage was performed between the JKWPKLP HIV line-listing database and the NAR. The deterministic (exact) matching method was employed to connect the records in two national databases using the unique identification number. In Malaysia, HIV is a notifiable disease by law, under Act 342: Prevention and Control of Infectious Disease (22). HIV cases were confirmed based on criteria stipulated in the national guideline—Case Definition of Infectious Diseases in Malaysia (23).

The NAR collected all mandatory notifications of PLHIV from all healthcare professionals nationwide. All confirmed cases were updated in the real-time NAR database and the HIV/STI/Hepatitis C Unit at the state level monthly audited them every month.

The JKWPKLP HIV line-listing database stored the medical records of PLHIV managed in JKWPKLP government health clinics. The database template was set up by the HIV/STI/Hepatitis C Sector, Ministry of Health. According to the guideline by the Malaysian Society of HIV Medicine (7), baseline investigation should be taken for initial evaluation of PLHIV at the entry to care which includes CD4 count and other investigations to seek the presence of any HIV co-infection. All information was entered into the registry by the appointed medical assistant or staff nurses of the respective HIV team for each of the health clinics. The HIV team consisting of a Family Medicine Specialist (FMS), medical officer and medical assistants or staff nurses were provided regular yearly training by the HIV/STI/Hepatitis C Unit at the state level. The JKWPKLP HIV line-listing database was updated monthly and verified by the respective FMS at the end of each month before being sent for a half-yearly audit at the state level.

### Study Variables

Thirteen variables were extracted from these two national data sources for further analysis. Outcome variable refers to successful HIV treatment (undetectable viral load < 200 copies/mL after 1-year antiretroviral therapy (ART) initiation, coded as Yes/No (5, 6, 8, 9). Data on the 12 independent variables were taken at the time of diagnosis and selection was made based on previous literature and clinical importance in managing PLHIV.

Of the 43 total variables from the JKWPKLP HIV line-listing database, 10 variables were extracted, including: i) age (years old); ii) gender (male/female); iii) HIV risk exposure (IVDU/homosexual, heterosexual); iv) baseline CD4 counts (cells/mm^3^); v) tuberculosis (TB) co-infection (yes/no); vi) Hepatitis C co-infection (yes/no); vii) Hepatitis B co-infection (yes/no); viii) healthcare services; ix) timing of ART initiation (days) and x) HIV viral load after 1 year of ART (copies/mL). Three more variables: i) education level of PLHIV (primary/secondary/tertiary); ii) employment status of PLHIV (yes/no) and iii) marital status of PLHIV (married/divorced/single) were extracted from the 76 total variables of NAR.

‘Healthcare service’ referred to the establishment of STIFC (coded as yes/no). STIFC was initiated in 2015 by MOH to facilitate HIV treatment among key populations by offering fast-track services to this population (19). In the STIFC, the PLHIV can bypass the registration process and receive their follow-up in a dedicated room with a certified HIV counsellor.

### Data Management

**Figure 2:**
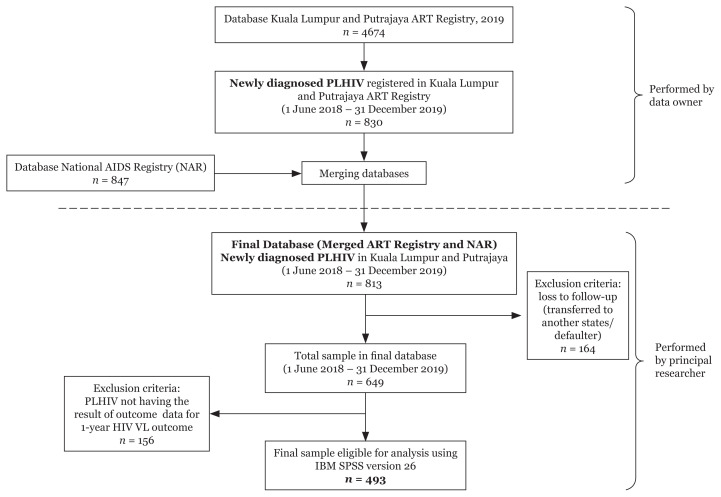
Flowchart of data extraction

Figure 2 depicts the process of data extraction which incorporates both national data sources in the form of Microsoft Excel Open XML Spreadsheet (xlsx) format. Data cleaning was performed using the final database (*n* = 813). Of 813 total samples, 493 (60.6%) were found eligible for analysis after applying the inclusion and exclusion criteria. The final database (*n* = 493) was imported into IBM SPSS version 26.0 for screening of missing data and further statistical analysis. The dataset contained missing values ranging from 0.6% to 8.7%. Little’s Missing Completely at Random (MCAR) test was used to determine the pattern of missingness. As the analysis showed a pattern of missing not at random, mean imputation was performed to replace the missing value of baseline CD4 count. Missing values for other variables reporting a percentage of < 5% were ignored (24).

In this study, confidentiality was maintained and all identifying information was deleted from the database to preserve the anonymity of participants. Further, data were kept secured and were made available only to the researcher. No personal information was disclosed, and the participants could not be identified from the research findings.

### Statistical Analysis

Data were then analysed using the IBM SPSS version 26.0 (25). The HIV treatment success rate (proportion of subjects having successful HIV treatment in the present study) was calculated by using the formula as followed:


$$\text{Rate of successful HIV treatment}=\frac{\text{Number of PLHIV with successful HIV treatment}}{\text{Total number of newly diagnosed PLHIV from June }2018\;\text{until December }2019}$$

Estimation of the rate of successful HIV treatment (95% confidence interval [CI] for the proportion interval) was calculated by using the following formula:


$$95\%\;\text{confidence interval }(\text{CI})=p\pm z*\sqrt{\frac{p(1-p)}{n}}$$

Analysis was performed to describe the overall characteristics of PLHIV in Kuala Lumpur and Putrajaya and those based on HIV treatment outcomes. The mean (standard deviation [SD]) was presented for continuous variables, while frequency (%) was represented for categorical data. The distribution of PLHIV was presented in general and based on their treatment outcomes. Descriptive and univariable analyses (independent *t*-test, chi-square test for homogeneity and continuity correction test) were used to compare the distribution. Univariable (simple logistic regression) and multivariable (multiple logistic regressions) analyses were performed to estimate the crude and adjusted effect of the independent variables of successful HIV treatment. Variables were selected into the multiple logistic regression model based on the level of statistical significance of a *P*-value (> 0.25) and clinical importance (26). Variable selection was done using the Backward Likelihood Ratio (LR) method. Next, the preliminary model was tested for linearity in the logit, interaction, and multicollinearity. Model fitness was verified by using Hosmer-Lemeshow goodness-of-fit test, classification table and receiving operating characteristic (ROC) curve. The strength of association between each factor and outcome measure was presented as crude/adjusted odds ratio (OR), 95% CI and their corresponding *P*-values. The level of significance for the statistical test was 0.05.

## Results

A total of 493 PLHIV was recruited for the study. The rate of successful HIV treatment was 92.2% (95% CI: 89.8%, 94.6%). Table 1 presents the characteristics of PLHIV who were predominantly male (474/493, 96.0%) and the mean (SD) age was 30 (8.1) years old. The mean (SD) timing for ART initiation was 54 (58.8) days, ranging from 0 to 113 days. Both groups of HIV treatment outcomes had comparable individual and disease-related characteristics.

Table 2 shows the estimated regression coefficient (standard error), Wald statistics (degree of freedom), crude odds ratio (95% CI) and corresponding *P*-value using simple logistic regression analysis. When unadjusted, three variables (i.e. HIV risk exposure, the timing of ART initiation and healthcare service) showed significant association with successful HIV treatment at a *P* < 0.05.

Subsequently, seven clinically important variables which demonstrated significant associations at the level of significance *P* < 0.25 in the simple logistic regression analysis were selected for the multivariable analysis. In the preliminary main multiple logistic regression model, the timing of ART initiation did not fulfil the linearity in the logit assumption (which was conducted by plotting the midpoints of its quartile groups and regression coefficients to observe the linearity); hence, the variable was categorised into four groups based on its quartile groups. The multiple logistic regression (final model) revealed two significant determinants, including the timing of ART initiation within 6 days to 30 days (AOR = 3.94; 95% CI: 1.32; 11.70) and establishment of STIFC (AOR = 3.40; 95% CI: 1.47, 7.85) (Table 3). Gender, education level, HIV risk exposure and co-infections of tuberculosis and Hepatitis C were the non-significant variables.

## Discussion

This retrospective cohort study attempted to determine the success rate of HIV treatment and its determinants among PLHIV in Kuala Lumpur and Putrajaya. Three key findings were: i) the rate of successful HIV treatment was reported at 92.2% (95% CI: 89.8%, 94.6%); ii) the key characteristics of PLHIV in Kuala Lumpur and Putrajaya were that they were in the young age group (in their thirties on average), predominantly male and homosexual/heterosexually exposed and iii) the significant determinants of successful HIV treatment were the timing of ART initiation and healthcare service (establishment of STIFC).

Our study data demonstrated that approximately 90% of newly diagnosed PLHIV had successfully achieved undetectable HIV viral load after 1 year of ART. The result seems to be in congruence with a previous study conducted in the United States, which observed a success rate of 91% (undetectable HIV viral load < 200 copies/mL) at 6 months and 1-year post ART initiation (17). On the other hand, our findings showed a higher success rate of HIV treatment compared to finding from a study in the United Kingdom (18), which reported that only 66.6% of their PLHIV on treatment achieved undetectable HIV viral load. In recent years, various initiatives have been introduced by the Malaysian government in ensuring the PLHIV attain a successful outcome. These measures include an upscale number of health facilities, training of paramedic HIV counsellors, strengthening the engagement with non-governmental organisations, and stigma and discrimination reduction programmes (19). Therefore, the high rate of successful HIV treatment in the current study was postulated to be credited to these initiatives, which have created a conducive and supportive environment for the PLHIV to seek proper medical attention and medication.

We also found that PLHIV’s average age of diagnosis was 30 years old. This is in concordance with other literature which reported similar findings, with the average age ranging from 30 years old to 35 years old during the initial HIV diagnosis (11, 12, 14, 27, 28). These findings were corroborated by the national reports based on the yearly surveillance on HIV, whereby almost half of new HIV cases in 2019 were found in the 20-years-olds–29-year-olds (29). In the natural history of HIV, the time of infectivity to diagnosis could extend to 5 years (30). Hence, HIV transmission in the key population can be as early as ≤ 25 years old. The preponderance of HIV in the young age group can perhaps be attributed to the lack of knowledge and awareness regarding HIV infection by young adults and adolescents (31).

Furthermore, male PLHIV constituted most of the study population. This is similar to the previous findings in the United States (18) and Vietnam (16) in which predominant male PLHIV with 96% and 66% of their study population, respectively. The higher proportion of male PLHIV in our study could be attributed to the high proportion of homosexual PLHIV, which contributes the most to the current HIV epidemic in Malaysia (19). Also, 99% of the new HIV cases involved sexual exposure, which is consistent with the national reports (19) and other studies (11, 15). The recent Integrated Bio-Behavioural Surveillance Survey 2017 highlighted that only 60% of homosexual PLHIV practice safe sex, in contrast to a higher proportion of safe injecting practices which remained > 80% among the people who inject drugs (PWID) in Malaysia (32). On account of this survey, HIV infections were more prevalent among the homosexual PLHIV as compared to PWID in Malaysia.

We would like to highlight the significant treatment-related determinants in predicting successful HIV treatment outcomes among PLHIV. Based on the global policy, WHO recommends initiating ART as early as possible in managing PLHIV to mitigate the risk of disease progression and prevent HIV transmission (5). We also document that the initiation of ART as early as 6 days–30 days from the diagnosis demonstrated significantly higher odds to attain successful HIV treatment as compared to PLHIV with delayed ART initiation (> 90 days). Our findings corroborate findings of a previous study conducted in San Diego, U.S. in 2016, in which 79% of those who initiated ART within 30 days of diagnosis achieved viral load suppression as early as week 12 (33). Moreover, a recent clinical trial found that PLHIV receiving early initiation of ART had lower rates of complication (1.8%; 0.60 events per 100 person-years) as compared to delayed initiation of ART (4.1%; 1.38 events per 100 person-years) (34). ART is the leading treatment of HIV, which has shown to be beneficial in the treatment of PLHIV by leading them to rapid immunologic and virologic recovery (4–6). Therefore, the present findings emphasised the importance of early initiation of ART in the management of PLHIV in Malaysia.

Additionally, PLHIV receiving care at STIFC were found to have higher odds of achieving successful HIV treatment compared to those under follow-up in conventional clinics (non-STIFC). Congruently, a recent rapid systematic review reported viral suppression exceeded 90% in 11 out of 21 differentiated service delivery models with a comparison to conventional care (35). STIFC was introduced in 2015 in Malaysia to ensure that the appropriate treatments remain accessible to the key populations, ensuring delivery of the provided care services via a friendly and stigma-free environment (36). Previous studies have shown that health facilities with limited differentiated service delivery were identified as one of the barriers to implementation of ART delivery, which can be attributed to self and community-related stigma and insufficient training of health care workers in such health facilities (37). An innovative and customised service delivery approach such as the STIFC, in which early identification, greater treatment adherence through comprehensive ART counselling and regular testing in an enabling environment are made possible, leads to a better chance for the PLHIV to have better treatment outcomes.

Besides suggesting the reinforcement of early ART initiation and implementation of differentiated health service delivery (e.g. the establishment of STIFC) for PLHIV all over Malaysia, the authors urge the health education and promotion activities to be targeted at teenagers or young adult population, e.g. introducing a validated and comprehensive school-based HIV education modules, in order to expose them to the gravity of HIV infection and HIV avoidance. The UNAIDS has recently highlighted several key preventive strategies, involving: i) delivery by trained adult facilitators; ii) multisession programmes; iii) curricula that include skill- and knowledge-building activities and iv) programmes tailored to the social context (38). More investigations may be needed in the future to look for the potential contributing factors for the delayed initiation of ART and its complications (39). Future work could embark on applying a qualitative approach in evaluating the STIFC establishment and how it affects successful HIV treatment outcomes, by exploring how stigmatisation influences the health-seeking behaviour of PLHIV.

The retrospective cohort study design facilitates better estimation of the predictive effect of successful HIV treatment compared to the usual cross-sectional study (40). To the best of our knowledge, we report the latest original study assessing the determinants of successful HIV treatment using a smaller cut-off point of HIV viral load, based on recent recommendations (6). Notably, the usage of smaller cut-off points for HIV viral load in this study could lead us to achieve the universal TasP strategy for PLHIV (6). Since this study used an HIV viral load of < 200 copies/mL, it is considered a meticulous determinant of viral load with a targeted approach rather than a broader definition of HIV viral load suppression (HIV viral load < 1000 copies/mL). We recruited PLHIV with various HIV staging, thereby minimising the selection bias and preserving the internal validity of the study (41).

The present study is restricted by the shortcomings of the registries (i.e. the source of secondary data). Hence, certain variables like adherence and drug resistance that might be contributing factors to virologic failure were not readily available for investigations. This circumstance will lead to the issue of residual confounding (42). Despite the acceptable applicability and generalisability of the present study as we analysed a large number of participants in a multicentre analysis, this health clinic-based study precludes the representation of PLHIV receiving care in hospital settings or private clinics. Information on non-Malaysian PLHIV was also not considered. Furthermore, PLHIV coming from resource-poor-health facilities, especially in rural areas or clinics without the establishment of STIFC (19), may not be represented in this study. This finding might impose limited applicability when applied to states not providing STIFC facilities.

## Conclusion

Achieving and maintaining an undetectable HIV viral load is one of the primary objectives in HIV treatment to prevent further transmission, especially among PLHIV whose HIV infections are transmitted via sexual mode. The JKWPKLP is on track towards achieving 95% of total PLHIV on treatment to have a successful treatment outcome at 1-year post ART initiation, with viral load suppressed < 200 copies/mL. A lower cut-off point of viral load suppression shall be advocated in the management of PLHIV (200 instead of 1,000 copies/mL), aligning with the concept of U = U. Overall, early ART initiation and the establishment of differentiated health service delivery (e.g. STIFC) in managing PLHIV will aid the clinicians and policymakers to minimise new HIV infections, thus achieving WHO goal to end AIDS by 2030.

## Figures and Tables

**Table 1:** Characteristics of PLHIV overall and according to the HIV treatment outcome (*N* = 493)

Variables	HIV treatment outcome			*P-value

	Total (*N* = 493) mean (SD)/*n* (%)	Successful (*N* = 454) mean (SD)/*n* (%)	Non-successful (*N* = 39) mean (SD)/*n* (%)	
Individual-related characteristics				
Age (years)	30 (8.1)	30 (8.2)	31 (6.6)	**0.450** a
Gender				
Female	19 (3.9)	16 (3.5)	3 (7.7)	**0.387** b
Male	474 (96.1)	438 (96.5)	36 (92.3)	
Education level				
Primary/Secondary education	163 (34.5)	149 (34.1)	14 (38.9)	**0.561** c
Higher education	310 (65.5)	288 (65.9)	22 (61.1)	
Employment status				
Not employed	32 (6.8)	30 (6.9)	2 (5.7)	**0.900** b
Employed	436 (93.2)	403 (93.1)	33 (94.3)	
Marital status				
Married	43 (9.1)	42 (9.6)	1 (2.8)	**0.288** b
Single/Divorced or Widowed	432 (90.9)	397 (90.4)	35 (97.2)	
HIV risk exposure				
Non-sexual (IVDU)	5 (1.0)	3 (0.7)	2 (5.1)	**0.067** b
Sexual (Homo/Heterosexual)	485 (99.0)	448 (99.3)	37 (94.9)	
Disease-related characteristics				
Baseline CD4 level (cells/mm^3^)	325 (230.1)	322 (210.2)	363 (391.3)	**0.538** a
Tuberculosis co-infection				
Yes	55 (11.2)	48 (10.6)	7 (17.9)	**0.255** b
No	438 (88.8)	406 (89.4)	32 (82.1)	
Hepatitis B co-infection				
Yes	25 (5.1)	23 (5.1)	2 (5.1)	**0.900** b
No	468 (94.9)	431 (94.9)	37 (94.9)	
Hepatitis C co-infection				
Yes	2 (0.4)	1 (0.2)	1 (2.6)	**0.370** b
No	491 (99.6)	453 (99.8)	38 (97.4)	
Treatment-related characteristics				
Timing of ART initiation (days)	54 (58.8)	51 (53.4)	88 (97.3)	**0.026** a
Healthcare service				
Non-STIFC	93 (18.9)	77 (17.0)	16 (41.0)	< 0.001^c^
STIFC	400 (81.1)	377 (83.0)	23 (59.0)	

Notes: ART = antiretroviral therapy; CD4 = cluster of differentiation 4; HIV = Human Immunodeficiency Virus; IVDU = intravenous drug user; PLHIV = people living with HIV; SD = standard deviation; STIFC = Sexually Transmitted Infection Friendly Clinic;

a Independent *t*-test/

b continuity correction (Yates’ correction);

c Chi-square test for homogeneity;

* level of significance set at 0.05

**Table 2:** Variables associated with successful HIV treatment using simple logistic regression (*N* = 493)

Variables	B (SE)	Wald (df)	Crude OR (95% CI)	*P-value
Age (years old)	−0.015 (0.019)	0.571 (1)	0.986 (0.949, 1.023)	**0.450**
Gender				
Female			1	
Male	0.825 (0.653)	1.597 (1)	2.281 (0.635, 8.197)	**0.206**
Education level				
Primary education			1	
Secondary education	0.884 (0.926)	0.788 (1)	2.420 (0.388, 15.095)	**0.342**
Higher education	1.099 (10.923)	1.082 (1)	3.000 (0.481, 18.706)	**0.237**
Employment status				
Not employed			1	
Employed	−0.206 (0.752)	0.075 (1)	0.814 (0.186, 3.558)	**0.785**
Marital status				
Married			1	
Divorced or widowed	−1.090 (1.368)	1.112 (1)	0.336 (0.022, 5.052)	**0.427**
Single	−0.820 (0.873)	1.608 (1)	0.441 (0.079, 2.463)	**0.349**
HIV risk exposure				
IVDU			1	
Homosexual	2.370 (0.938)	6.385 (1)	10.696 (1.702, 67.218)	**0.012**
Heterosexual	1.325 (0.958)	1.913 (1)	3.762 (0.576, 24.587)	**0.167**
Baseline CD4 level (cells/mm^3^)	−0.001 (0.001)	1.084 (1)	0.999 (0.998, 1.001)	**0.298**
Tuberculosis co-infection				
Yes			1	
No	0.615 (0.444)	1.918 (1)	1.850 (0.775, 4.420)	**0.166**
Hepatitis B co-infection				
Yes			1	
No	0.013 (0.757)	0.000 (1)	1.013 (0.230, 4.465)	**0.986**
Hepatitis C co-infection				
Yes			1	
No	2.478 (1.424)	3.028 (1)	11.921 (0.731, 194.379)	**0.082**
Timing of ART initiation (days)	−0.007 (0.002)	11.806 (1)	0.993 (0.989, 0.997)	**0.001**
Healthcare service				
Non-STIFC			1	
STIFC	1.226 (0.349)	12.350 (1)	3.406 (1.719, 6.747)	< 0.001

Notes: ART = Antiretroviral therapy; B = unstandardised regression weight; CD4 = cluster of differentiation 4; CI = Confidence interval; df = degree of freedom; HIV = Human Immunodeficiency Virus; IVDU = Intravenous Drug User; OR = odds ratio; SE = Standard error; STIFC = Sexually Transmitted Infection Friendly Clinic;

* level of significance set at 0.05

**Table 3:** Determinants of successful HIV treatment in Kuala Lumpur and Putrajaya using multiple logistic regression analysis (*N* = 493)

Variables	B (SE)	Wald (df)	AOR (95% CI)	*P-value
Timing of ART initiation				
> 90 days			1	
< 5 days	0.761 (0.706)	1.162 (1)	2.141 (0.536–8.547)	0.28
6 days–30 days	1.370 (0.556)	6.067 (1)	3.943 (1.323–11.699)	0.01
31 days–90 days	0.675 (0.446)	2.294 (1)	1.964 (0.820–4.720)	0.13
Healthcare service				
Non-STIFC			1	
STIFC	1.223 (0.427)	8.198 (1)	3.397 (1.471–7.848)	0.004

Notes: AOR = adjusted odds ratio; ART = Antiretroviral therapy; B = unstandardised regression weight; CI = confidence interval; df = degree of freedom; SE = Standard error; STIFC = Sexually Transmitted Infection Friendly Clinic;

* level of significance set at 0.05; Constant = −0.267; Backward LR method was applied; No multicollinearity and no interaction; Hosmer Lemeshow test, *P*-value = 0.903; Classification table 92.2% correctly classified; Area under Receiver Operating Characteristics (ROC) curve = 0.67
